# Total acoustic transmission in a honeycomb network empowered by compact acoustic isolator

**DOI:** 10.1038/s41598-023-28097-y

**Published:** 2023-01-16

**Authors:** Haixiao Zhang, Rong Li, Yu Bao, Xiaoli Liu, Yiwei Zhang

**Affiliations:** 1 School of Electrical and Information Engineering, Changzhou Institute of Technology, Changzhou, 213032 China; 2 MOE Key Laboratory of Modern Acoustics, Nanjing University, Nanjing, 210093 China; 3 Telecommunication and Networks National Laboratory, Nanjing University of Posts and Telecommunications, Nanjing, 210003 China; 4 School of Aviation and Mechanical Engineering, Changzhou Institute of Technology, Changzhou, 213032 China

**Keywords:** Acoustics, Mechanical engineering

## Abstract

In recent years, acoustic metamaterials have exhibited extraordinary potential for manipulating the propagation of sound waves. However, it has been a challenge to control the propagation of sound waves through arbitrary pathways in a network. In this work, we designed a compact three-port isolator that can produce giant acoustic nonreciprocity by introducing actively controlled CNT films to the device without altering the geometric symmetry of it. This concept is subsequently applied to construct a 4 × 7 honeycomb network, in which, total transmission of sound wave in arbitrary pathway can be slickly achieved. Unlike the acoustic topological insulator, which only supports total transmission of arbitrary pathway in the band gap, our method provides more degrees of freedom and can be realized at any frequency. This ability opens up a new method for routing sound waves and exhibits promising applications ranging from acoustic communication to energy transmission.

## Introduction

Acoustic metamaterials provide fascinating opportunities for artificially manipulating the propagation of sound waves^1–3^. At this rate, they have potential for applications in fields such as acoustic cloaking^4–7^, parity-time symmetry^8–12^, supertunneling^13–15^, and logic gates^16,17^. In recent years, how to control the propagation of sound waves through arbitrary pathway in a network has been a very challenging subject. Inspired by the electronic edge states occurring in topological insulators^18,19^, the concept of topological acoustics has been proposed^20^, and the phenomenon of disorder-free, one-way sound propagation has been experimentally demonstrated^21–25^. Besides, the combination of a symmetry-breaking, cross-shaped metamaterial comprising Helmholtz resonant cells and an eccentrically arranged square column can also lead to quasilossless acoustic transmission in an arbitrary pathway of a network^26^. It is worth noting that recent progress in a phononic metamaterial with a topologically protected elastic wave that is robust against scattering from discrete defects and disorders has been constructed^27,28^. In view of the correlation between elastic and sound waves, elastic metamaterials may also give rise to potential ideas for experimental realization of acoustic topology, and vice versa.

The aforementioned topologically protected edge states and reconfigurable topological one-way transmission for sound wave can be accomplished expediently by revising the geometry of the structure. However, the dispersion of a passive, linear time-invariant acoustic medium is bounded by Kramers-Kronig relations, limiting the spectral range of the target effective properties. Furthermore, the efficiency of passive acoustic metamaterials is most likely hindered by losses, as there is no compensation mechanism for the undesirable inherent visco-thermal dissipations. On account of this, there has been recently an intense surge of interest for active acoustic metamaterials, which could broadly enhance the real-world applicability of acoustic metamaterials by overcoming the challenges^29^. Active acoustic metamaterials have been widely put into use in various fields of acoustics and have played an important role, including acoustic gain^30^, parity-time symmetric acoustic metamaterials^31–33^, constant amplitude acoustic waves^34,35^, reconfigurable metasurfaces^36,37^, non-reciprocal meta-atoms^38–43^ and topological metamaterials with external drive^44–46^. This indicates that active acoustic metamaterials hold significant promise and value for applications requiring lossless transmission.

In this paper, we introduce the currently popular active acoustic material, carbon nanotube (CNT) film into a compact three-port acoustic device and achieve giant acoustic nonreciprocity by regulating the thermo-acoustic parameters of the films. The acoustic isolators are then organized into a 4 × 7 honeycomb network. Robust isolation allows us to arbitrarily control the sound wave propagation pathway in the honeycomb network and maintain the transmission coefficient of one at any frequency, as long as we effectively control the parameters of the introduced CNT films. The finite element simulation results fully confirm the validity of the above conclusions. We believe that total transmission concepts can enormously expand the engineering toolkit for modern acoustic devices and open up a versatile way to control the propagation of sound waves.

## Results and discussion

### Unbiased acoustic three-port device

**Figure 1:**
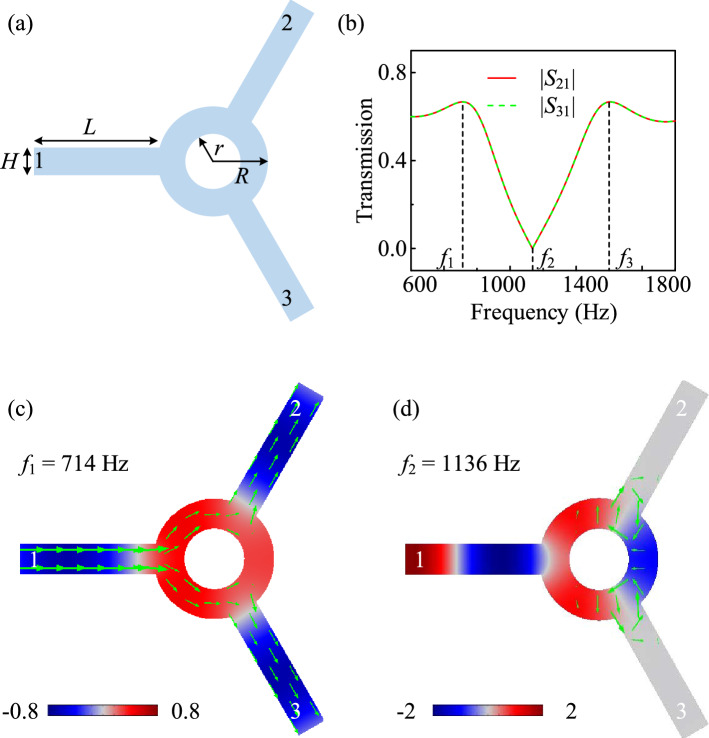
Schematic of an unbiased acoustic three-port device. (**a**) The geometry of the proposed device. (**b**) The amplitudes of the pressure transmission coefficients at ports 2 and 3 in the absence of extra bias. (**c**, **d**) Acoustic pressure field distributions inside the device at $f_1=714$ Hz (**c**) and $f_2=1136$ Hz (**d**). Green arrows represent the average acoustic power flow in (**c**, **d**).

We generally begin with an unbiased three-port device with three waveguides symmetrically placed at 120° intervals, and assume an acoustic wave incident from port 1. If nothing else, the counterclockwise (CCW) and clockwise (CW) modes of the sound wave are degenerate at any frequency. In order to investigate the behavior of it, we meticulously set the geometrical parameters of the device with waveguide width $H=5$ cm, length $L=22.5$ cm, and inner radii $r=5$ cm, outer radii $R=10$ cm of the ring cavity in Fig. 1a. The amplitudes of the pressure transmission coefficients at ports 2 and 3 in the absence of biasing control is shown in Fig. 1b. In this case, the transmission coefficients at the two output ports are identical due to symmetry. The unbiased cavity simply forms a power divider, which sends 4/9 of the power to each of the output ports, and the remaining 1/9 is reflected at the 1st resonance of $f_1=714$ Hz. At the decay mode of $f_2=1136$ Hz, both the transmission coefficients decrease to zero, indicating a power blocker. As the frequency increases, the transmission coefficients gradually grow until the 2nd resonance of $f_3=1608$ Hz, at which the amplitude transmission coefficients go back to 2/3.

To gain further insights into the response of the unbiased device, we show the pressure distributions for aforementioned two cases in Fig. 1c and d. In both cases, the modes are degenerate and evenly excited, resulting in a field distribution inside the cavity that is totally symmetric with respect to the axis of port 1. Ports 2 and 3, which are symmetrically placed, are therefore evenly excited, and the response is fully reciprocal. The average power flow, represented by the green arrows, is split evenly between the two output ports.

### Compact acoustic isolator

**Figure 2:**
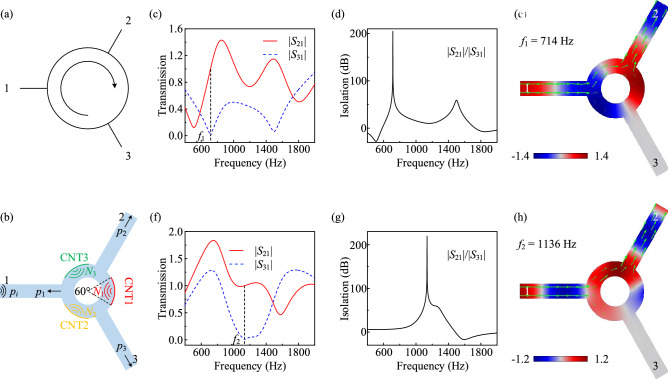
Compact acoustic isolator. (**a**, **b**) An isolator as an exception of a circulator, its schematic model (**a**) and its implementation by using three actively controlled CNT films stuck to the wall of the ring (**b**). Sound propagates from port 1 to 2, but not 3. (**c**) Case of optimal biasing control (Case I) for maximum nonreciprocity, with total transmission to port 2 and zero transmission to port 3 at $f_1=714$ Hz. (**d**) Isolation in decibels with $|S_{21}|/|S_{31}|$. (**e**) Acoustic pressure field distribution inside the optimally biased device. (**f**–**h**) Same as (**c**–**e**), but with the CNT film parameters of Case II at $f_2=1136$ Hz. Green arrows represent the average acoustic power flow in (**e**) and (**h**).

In this section, we demonstrate how to implement a compact acoustic isolator with a potential active acoustic material, in which, ports 2 and 3 have distinct responses despite the geometrical symmetry of the cavity (see Fig. 2a). The potential material, CNT film, has been utilized to act as acoustic gain in non-Hermitian topological whispering gallery^47^. We constructed such a device by attaching three CNT films of 60$^\circ$ arc length to the inner surface of the cavity directly opposite the waveguides and applying electrical actuations to them, as shown in Fig. 2b. On this basis, the pressure field inside the device can be obtained by the linear superposition of the incident wave ($p_i=1$ Pa) at port 1 and the waves generated by the CNT films, in which, various multifunctional three-port acoustic devices can be realized. Thus, the reflection pressure at port 1 ($p_1$), the transmission pressure at port 2 ($p_2$) and port 3 ($p_3$) motivated by the incident wave from port 1 and three actively controlled CNT films can be expressed as (see Fig. 2b)

1 $$\begin{aligned} \left[ \begin{matrix}p_1\\ p_2\\ p_3\end{matrix}\right] =\left[ \begin{matrix}p_r\\ p_t\\ p_t\end{matrix}\right] +\left[ \begin{matrix}t_o&{}t_a&{}t_a\\ t_a&{}t_o&{}t_a\\ t_a&{}t_a&{}t_o\end{matrix}\right] \left[ \begin{matrix}N_1\\ N_2\\ N_3\end{matrix}\right] . \end{aligned}$$

Here, $p_r$ and $p_t$ are the reflection and transmission pressures at port 1 and port 2 (3) when the incident wave of 1 Pa comes from port 1, which can be extracted from the simulation of COMSOL Multiphysics. Homoplastically, $t_o$ and $t_a$ are the outgoing pressures at the opposite and adjacent ports of a single motivated CNT film with 1Pa. In addition, $N_m=n_m\varphi _m$ ($m=1,2,3$) is the amplitude ratio ($n_m$) and phase difference ($\varphi _m$) of the corresponding CNT film to the sound source of 1 Pa at port 1.

After straightforward calculations, we obtain $N_m$ with

2 $$\begin{aligned} \left[ \begin{matrix}N_1\\ N_2\\ N_3\end{matrix}\right] =\left[ \begin{matrix}t_o&{}t_a&{}t_a\\ t_a&{}t_o&{}t_a\\ t_a&{}t_a&{}t_o\end{matrix}\right] ^{-1} \left[ \begin{matrix}p_1-p_r\\ p_2-p_t\\ p_3-p_t\end{matrix}\right] . \end{aligned}$$

Equation (2) presents a mathematical model for a three-port acoustic device with adjustable scattering properties, in which the active parameters ($N_m$) of the three CNT films are dependent on the targeted scattering properties of the device. In this model, $p_1=0$ ($S_{11}=p_1/p_i$) ensures that there is no reflected wave at port 1, and $p_3=0$ ($S_{31}=p_3/p_i$) indicates that there is no transmitted wave at port 3. At the same time, $|p_2|=1$ ($S_{21}=p_2/p_i$) guarantees unitary transmitted wave at port 2, thus constructing a three-port acoustic isolator integrally. This is quite different from the case in Ref.^40^, in which the authors introduce the acoustic circulator in a subwavelength meta-atom consisting of a resonant ring cavity biased by a circulating fluid. Thus, the resulting angular momentum bias splits the ring’s azimuthal resonant modes, producing giant acoustic nonreciprocity.

**Table 1:** Accessing the compact acoustic isolator.

Parameters of CNT films	Case I ($f_1=714$ Hz)	Case II ($f_2=1136$ Hz)
$n_1$	0.2434	0.4768
$\varphi _1$	− 1.3548	1.2986
$n_2$	0.2433	0.4768
$\varphi _2$	1.2260	2.9228
$n_3$	0.5684	0.6499
$\varphi _3$	− 0.0644	2.1107

The introduction of the CNT films provide rich degrees of freedom for the control of the three-port isolator, so that we can adjust the phase of the transmission wave at port 2 at liberty. We calculate the parameters of CNT films ($n_m$ and $\varphi _m$) in Case I ($f_1=714$ Hz) with $p_2=e^{-k_1L}$ and Case II ($f_2=1136$ Hz) with $p_2=e^{-k_2L}$ respectively, as exhibited in Table 1. Here, $k_{1,2}=2\pi f_{1,2}/c_0$ is the wave number at operating frequency $f_{1,2}$, and $c_0$ is the sound speed in air. Spontaneously, Fig. 2c shows the altered transmission spectrum when the device is appropriately biased with optimal actively control in Case I, with total transmission to port 2 and zero to port 3 at $f_1=714$ Hz. To quantify the performance of the realized device, the simulated isolation $|S_{21}|/|S_{31}|$ is shown as a function of the operating frequency in Fig. 2d. Around the optimal bias value, this device produces very large values of isolation, up to 200 dB. The pressure field distribution inside the optimally biased device has been shown in Fig. 2e, in which the mode splitting is perfectly balanced to produce an asymmetric field distribution with respect to port 1. Namely, an unitary of pressure field at port 2 created by constructive interference between the two modes, and conversely, through destructive interference, a null at port 3. In this scenario, the power flow is routed exclusively toward the output port on the left of the input, depending on the feeding port. However, when the feeding port is converted, the parameters of CNT films should also be adjusted accordingly.

A similar phenomenon can be achieved at another frequency of $f_2=1136$ Hz, with the parameters of CNT films presented in the right column of Table 1. In this way, Fig. 2f shows the altered transmission spectrum when the device is appropriately biased with optimal actively control in Case II, meanwhile Fig. 2g exhibits the isolation in decibels between ports 2 and 3. The pressure field distribution at this frequency can be seen in Fig. 2h, which confirms the aforementioned conclusion.

### Total acoustic transmission in a honeycomb network

**Figure 3:**
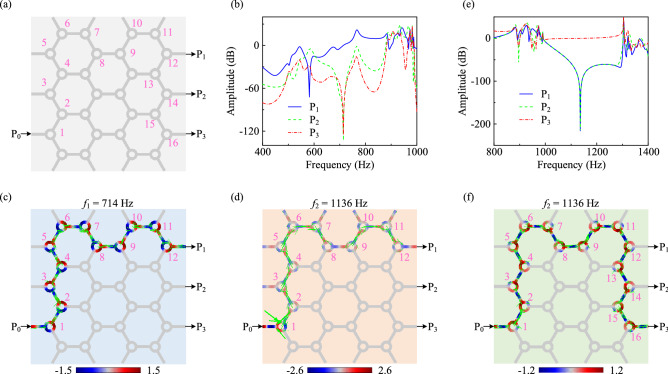
Total acoustic transmission in a honeycomb network. (**a**) A honeycomb network consisting of 4 × 7 compact acoustic isolators. The purple numbers are the indexes of the stand-by isolators. (**b**) Pressure amplitudes in decibels at ports P1, P2, and P3 with isolators 1–12 empowered in Case I. (**c**, **d**) Acoustic pressure distributions in a 12-unit pathway of the honeycomb network at $f_1=714$ Hz (**c**) and $f_2=1136$ Hz (**d**) in Case I. (**e**) Pressure amplitudes in decibels with isolators 1-16 empowered in Case II. (**f**) Acoustic pressure distribution in a 16-unit pathway of the honeycomb network at $f_2=1136$ Hz in Case II. Green arrows represent the average acoustic power flow in (**c**), (**d**) and (**f**).

Such a choreographed isolator provides a potential mentality for wave propagation along arbitrary pathway, as do acoustic topological insulators. However, this strategy gets rid of the limitation of structural band gap and can realize total transmission in arbitrary pathway at any frequency. In order to demonstrate the property visually, we build a honeycomb network, comprising 4 × 7 compact isolators with the size of 2.65 × 2.65 m^2^. In the simulation, we extract the pressure amplitude at three exit ports P1, P2, and P3 on the honeycomb network (P0 is the entrance port), as marked by the black arrows in Fig. 3a.

Fig. 3b exhibits the pressure amplitudes at ports P1, P2, and P3 with compact isolators 1–12 empowered becomingly in Case I, in which, an exceptionally significant property of acoustic transmission can be observed at $f_1=714$ Hz. On this occasion, an unitary transmission at port P1, and zero transmissions at the other two ports (P1 and P2) can be faultlessly demonstrated, achieving arbitrary control of the acoustic wave pathway. In Fig. 3c, the simulated amplitude distribution unequivocally shows the combination of both counterclockwise (1, 2, 4, 8, 9, 12) and clockwise (3, 5, 6, 7, 10, 11) modes, completing the regulation of sound waves along arbitrary pathway. If we change the frequency of incident wave without adjusting the CNT film parameters, we will lose the expected result, as shown in Fig. 3d. In this scenario, there are outgoing waves at the isolation port of each boundary unit, and the outgoing wave amplitude of port P1 is not unitary, indicating that the sound wave is no longer propagating along the designed pathway. However, the absence of outgoing waves at ports P2 and P3 comes from the transmission isolation of the unit itself at $f_2=1136$ Hz (see Fig. 1d).

The specific pathway of sound transmission and the corresponding excitation mode are adaptive. In Fig. 3e, we plot the pressure amplitudes at ports P1, P2 and P3 with isolators 1–16 empowered precisely in Case II. An unitary transmission at port P3, and zero transmissions at ports P1 & P2 can be observed at $f_2=1136$ Hz as expected. Indeed, the simulated amplitude distribution unequivocally shows the regulation of sound waves along designed pathway in Fig. 3f. Pathway control at different frequencies requires both isolator regulation and excitation of them in the designed pathway, which also provides more freedoms for the device design.

## Conclusions

In conclusion, we design an acoustic isolator by introducing actively controlled CNT films, which has no reflection at the input port, but shows giant nonreciprocity at two output ports. The results are well demonstrated at two typical frequencies, including the 1st resonance of $f_1=714$ Hz and the decay mode of $f_2=1136$ Hz. We then combine 28 independent isolators into a 4 × 7 honeycomb network in order and achieve total transmission of sound waves in arbitrary pathways at the two frequencies mentioned above. The finite element simulation results fully confirm the validity of the above conclusions. Distinguish from topologically robust sound propagation in an angular-momentum-biased graphene-like network^45^, our strategy has a wider frequency band and is more efficient. On the basis of these outstanding properties, we can envision unprecedented potential for routing sound waves achieving excellent propagation characteristics. We believe that total transmission concepts can enormously expand the engineering toolkit for modern acoustic devices and open up a versatile way to control the propagation of sound waves. Additionally, our findings significantly facilitate the experimental realization of acoustic wave propagation and is of fundamental importance in a wide range of acoustics, optics, and engineering applications.

## Methods

The numerical results presented in this paper were calculated using the commercial finite-element-method simulation software COMSOL Multiphysics. In the pressure-field calculations of the system, physical models were established and analyzed in the pressure acoustic module, including the detailed structures with actual geometric dimensions. The boundaries of the waveguides and ring were modelled as hard-wall boundary conditions. The parameters used for air were the mass density $\rho _0=1.21$ kg/m^3^ and sound speed $c_0=344$ m/s. Three CNT films of 60° arc length were attached to the inner surface of the cavity directly opposite the waveguides, as shown in Fig. 2b, and the thickness of one CNT film was $d=0.01$ cm. The mass density and heat capacity of CNT film were set to be $\rho =1400$ kg/m^3^ and $c_p=500$ J/kg K. The CNT film regions were then set as the Heat Sources, and the thermal radiation powers per unit volume (TRPPUV) of the heat sources were set as $q_m=N_mq_0$ according to the data in Table 1. Here $q_0$ is the TRPPUV when the near-field radiation sound pressure is 1 Pa, and its value can be obtained by simulation. The calculation domain is terminated by radiation boundary conditions that also includes the incident field.

## Data Availability

The datasets used and/or analysed during the current study available from the corresponding author on reasonable request.
